# Telemedicine as an Approach to the Mental Health of Healthcare Workers in Angola

**DOI:** 10.3390/ijerph22040565

**Published:** 2025-04-04

**Authors:** Edmilson Serra, Teresa Magalhães

**Affiliations:** 1 Sagrada Esperança Clinic, Av. Murtala Mohammed, 298, Luanda, Angola; 2 NOVA National School of Public Health, NOVA University Lisbon, 1600-560 Lisbon, Portugal; 3 NOVA National School of Public Health, Public Health Research Centre, Comprehensive Health Research Center, CHRC, REAL, CCAL, NOVA University Lisbon, 1600-560 Lisbon, Portugal

**Keywords:** telepsychiatry, occupational mental health, healthcare professionals, Angola

## Abstract

Introduction: African healthcare professionals face significant mental health challenges; therefore, telemedicine can overcome geographical barriers and improve access to mental healthcare. Objective: The objective of this study was to identify the key factors influencing the adoption of telemedicine as a tool to support healthcare workers’ mental health in an Angolan healthcare network and develop a telemedicine model tailored to this context. Methodology: This mixed-method study collected quantitative data from a questionnaire applied to healthcare workers (n = 275), which were analyzed using descriptive statistics and logistic regressions. Qualitative data were obtained through structured interviews (n = 5) with psychologists and psychiatrists, and analyzed using thematic analysis in MAXQDA (Version 2022, VERBI Software, Germany), to identify recurring patterns and themes. The data were triangulated to ensure the rigor and consistency of the findings. Participation was anonymous and voluntary, and informed consent was obtained from all participants. Results: Previous experiences with mental health consultations, perceptions of teleconsultations, and doctor–patient relationship were significant in influencing telemedicine adoption by workers. The thematic analysis revealed four themes: perception and ease of telemedicine use, intention to use, and the perception of mental health in Angola. The key adoption factors for providers included project feasibility, management support, training, payment policies, and adherence to legal, ethical, and deontological standards. Conclusions: The adoption of telemedicine for occupational mental health requires culturally adapted interventions and compliance with technological and data protection standards. Hospital management must address infrastructure challenges and mental health perceptions, and implement sustainable strategies that promote occupational well-being within the organization.

## 1. Background

Occupational mental health is a continuous and dynamic state of internal balance between job demands and the mental resources available to manage the occupational environment [1,2]. The COVID-19 pandemic exacerbated workplace demands and significantly impacted the mental health of healthcare professionals, resulting in high prevalence rates of depression, anxiety, post-traumatic stress, insomnia, and substance abuse [3].

Healthcare workers in Africa experienced mental health disorders [4,5,6,7], with additional challenges such as staff shortages, underfunding, and inadequate infrastructure [8,9,10]. Evidence highlights that nurses, women, and workers with less experience were particularly affected by mental health disorders during the pandemic [5,11]. Additionally, high rates of mental health issues were reported in countries like Nigeria, South Africa, Egypt, and Kenya [12,13,14,15].

Despite the existing gap in the literature on healthcare professionals’ mental health in Angola, there remains a lack of focus on developing and implementing specific interventions tailored to this context. Research by Miguel (2022) [16], conducted in a public hospital in the southern region of the country, reported significant psychological distress among Angolan healthcare professionals even before the first confirmed cases of COVID-19. There is a pressing need for mental health support interventions at national, organizational, and individual levels.

Angola faces a critical shortage of mental health professionals, with only 0.06 psychiatrists and 0.18 psychologists per 100,000 inhabitants [17,18], alongside a healthcare workforce far below the World Health Organization (WHO) standards [19]. Poor working conditions, inadequate remuneration, and professional undervaluation represent significant psychosocial risks, especially for public-sector nurses [20,21].

### 1.1. Impact on Healthcare Professionals’ Mental Health

Healthcare professionals are particularly vulnerable to mental disorders such as burnout, depression, anxiety, and substance abuse [22,23,24], with post-traumatic stress disorder (PTSD) emerging as a more recent concern [25]. The COVID-19 pandemic exacerbated these conditions, increasing tendencies toward suicide, sleep disturbances, and acute stress [11,26,27,28,29,30].

These mental health challenges impact not only workers, but also healthcare organizations and systems. Burnout, for instance, is associated with reduced patient safety, lower quality of care, and increased medical errors [31,32,33]. Presenteeism, defined as the condition in which professionals continue to work despite being unwell, reduces productivity may compromise care quality and is associated with burnout [34,35]. Depressive disorders contribute to absenteeism [36], presenteeism, and turnover, which further affect the quality of care [37,38]. Additionally, substance abuse increased during the pandemic [39,40], with significant prevalence in certain African countries [41]. These findings underline the importance of prioritizing mental health management in healthcare organizations, particularly in the post-pandemic context.

### 1.2. Digital Transformation and Healthcare Professionals’ Mental Health—Telemedicine as a Resource for Angola

The COVID-19 pandemic accelerated the adoption of digital technologies in healthcare, particularly telemedicine, to address increasing mental health demands and resource shortages [42,43]. Telemedicine extends beyond technology adoption, aiming to add value, improve access, optimize processes, and enhance the patient–provider relationship [44]. Within this framework, telepsychiatry—a branch of telehealth—offers reliable and effective mental health care, whether conducted synchronously or asynchronously [45,46].

Despite its advantages, digital transformation poses ethical and operational challenges, such as privacy concerns, data protection, the need for tailored infrastructure as well as scientific, clinical, and regulatory challenges [47]. Digital interventions, ranging from smartphone apps to computer-based therapies, have proven effective in promoting the accessibility and personalization of care [45,48,49]. For example, during the pandemic, initiatives like Malaysia’s Psychological First Aid protocol via WhatsApp [50] and Mount Sinai’s symptom monitoring app in the United States (USA) [51] expanded mental health care access for healthcare professionals. Similarly, digital applications in Spain and the United Kingdom (UK) demonstrated reductions in anxiety and depression, though further studies are needed to evaluate their long-term efficacy [52,53].

In Africa, digital health innovation is growing, with 14% of initiatives related to telemedicine [54]. However, the adoption of telepsychiatry remains limited due to infrastructural and cultural challenges [55]. In Angola, recent digital projects have focused on healthcare service delivery and information systems, but have not fully explored telemedicine for mental health [54]. A prominent private healthcare network in Angola, with a robust digital infrastructure and experience in teleconsultations during the pandemic, is well-positioned to lead advancements in telemedicine.

The integration of telemedicine into this network could address significant barriers, such as limited access to mental health care and geographical disparities. This study contributes to filling a critical gap in the literature by exploring telemedicine’s role in supporting healthcare professionals’ mental health in Angola. Regarding studies on telemedicine, questionnaires are frequently used as assessment tools. However, few studies focus on the factors influencing the acceptance and implementation of telemedicine [56]. Quantitative or qualitative studies are often directed at healthcare providers and patients. For example, an exploratory review highlighted that, out of the 53 analyzed articles, 49% assessed satisfaction, 34% usability, 11.5% acceptance, and only 2% the implementation of telemedicine services [57]. In this context, a mixed-methods study could represent an opportunity in the Angolan setting to better explain the adoption of telemedicine.

### 1.3. Contextualization and Evaluation of Workers’ Mental Health

A prominent private healthcare network in Angola plays a significant role in the country’s healthcare landscape, operating a central tertiary hospital in Luanda and 23 partner clinics across 16 provinces. Employing approximately 3000 workers, the network provides primary and secondary care, with referrals to its central facility.

The network includes a structured mental health circuit comprising a medical center and a Social Support Office. The medical center offers general and occupational health consultations, with referrals to mental health services based on clinical criteria. The Social Support Office provides psychological support, self-help groups, and educational lectures. However, from 2020 to 2024, only 39 psychology and 58 psychiatry consultations were conducted for workers, raising questions about the perception of these services among professionals and whether the circuit adequately addresses the needs of healthcare workers in geographically distant units. These observations underscore the necessity of implementing a specific occupational mental health program to ensure support across the network. In this context, telemedicine emerges as an opportunity to expand access to and the coverage of mental health care.

Although Angola has general mental health legislation, the Angolan General Labor Law does not include provisions for occupational mental health, and national policies lack targeted strategies for healthcare workers [58]. This legislative gap further emphasizes the importance of developing structured programs tailored to the needs of healthcare professionals within the private healthcare network. Most African countries do not include stress or related mental disorders in their national lists of occupational diseases [59].

This study aims to address the gap in the literature on digital mental health interventions in Angola by identifying and exploring the key factors influencing healthcare workers’ predisposition to adopt telemedicine as a tool for mental health support. Additionally, it seeks to develop a telemedicine model tailored to the specific needs of this healthcare network. This research aligns with Sustainable Development Goals (SDGs), particularly SDG 3 (Good Health and Well-Being) and SDG 8 (Decent Work and Economic Growth).

## 2. Methods

This observational, cross-sectional study employed a mixed-methods approach, primarily quantitative, complemented by a qualitative method. The aim was to provide an integrated approach to the phenomenon under study: the predisposition to adopt telemedicine. The quantitative approach targeted workers who may receive care, while the qualitative approach focused on professionals providing mental health care. The objective was to achieve complementarity between methods for better clarification and explanation of the results. This integrated approach in the study provided both the robustness for descriptive statistics and the interpretative depth necessary to address the phenomenon under investigation within the network.

The quantitative component followed an observational design without researcher interference, using a deductive approach. Data were collected at a single time point through a questionnaire and analyzed descriptively. Additionally, logistic regressions were conducted to assess the strength and direction of associations between sociodemographic predictors and factors influencing telemedicine adoption. The qualitative component, framed as a case study within an interpretive paradigm, aimed to explore the subjective meanings and experiences of mental health professionals. Using a deductive approach, data were collected through interviews and analyzed thematically to provide insights into healthcare professionals’ perceptions regarding the adoption of telemedicine for mental health support.

### 2.1. Research Questions:

This study aims to respond to the following questions:

Q1—What are the main factors influencing the predisposition of healthcare workers, both at the main facility and partner units, in Angola, to adopt telemedicine as a potential approach for their mental health (quantitative approach)?

Q2—What are the perceptions of mental health professionals regarding telemedicine and its adoption as a tool to support the mental health of workers in a private healthcare network in Angola (qualitative approach)?

#### 2.1.1. Quantitative Method

This is an observational, cross-sectional, and descriptive study.

#### 2.1.2. Study Population and Sampling

The study population included all healthcare workers directly involved in the provision of patient care within the private healthcare network. This group encompassed professionals from diverse categories, such as physicians, nurses, laboratory and imaging technicians, physiotherapists, pharmacy technicians, and administrative staff. Workers with less than three months of experience in the network and external collaborators were not included.

Participants were drawn from three regions of Angola—the capital (Luanda), eastern, and southern regions—focusing on units with the highest number of healthcare workers. Sampling followed a snowball technique, where initial participants shared the survey with their professional networks, enabling broader participation. This snowball sampling method was chosen due to the challenges of obtaining a larger sample within the Angolan context. Additionally, the limited availability of baseline studies in the literature made it difficult to establish reference parameters for determining sample representativeness. One of the researchers contacted potential participants from the main facility and partner units via email and WhatsApp to share the questionnaire, and they, in turn, distributed it within their network of contacts.

#### 2.1.3. Information Sources and Data Collection

Data collection was conducted using a culturally adapted questionnaire distributed at the main facility and partner units of the private healthcare network during February and March 2024. Confidentiality and anonymity were maintained throughout the data collection process. Participants provided prior consent for participation and were free to withdraw at any time. The questionnaire was distributed and completed online, with an estimated completion time of 10 min, accessed via a link directing to the Google Forms platform.

### 2.2. Questionnaire and Cultural Adaptation

The questionnaire used for quantitative data collection among professionals in the private healthcare network in Angola was developed based on the instrument presented in the article “Telepsychiatry to Provide Mental Health Support to Healthcare Professionals during the COVID-19 Crisis”. The questionnaire used in this study did not evaluate indirect dimensions, and the items were intended solely for descriptive analyses without score attribution.

The original English questionnaire was culturally adapted to Portuguese, with a pre-test conducted on a sample of n = 32, where 96.6% of participants found the questionnaire easy to understand (further details on cultural adaptation are provided in Supplementary Materials File S1).

#### 2.2.1. Study Variables

The independent variables in the study include participants’ sociodemographic data (gender, age, professional category, years of work experience, and geographic area of the health unit). The dependent variables were determined by the question groups in the questionnaire. The final version of the survey is available in Supplementary Materials File S1.

#### 2.2.2. Analysis and Processing of Quantitative Data

Data analysis was performed using statistical methods with version 29 of the Statistical Package for the Social Sciences (SPSS). Descriptive statistics were calculated as absolute frequencies and percentages. Bivariate analyses were conducted using the Chi-square test, with Fisher’s exact test applied when necessary. Statistical significance was considered at a 5% level (*p* < 0.05).

A binary logistic regression was performed for variables with statistically significant associations. The codings (0—No; 1—Yes) of the dependent categorical variables corresponded to the respective dichotomous questionnaire responses. Category 1 was analyzed in relation to category 0. Models were considered valid when statistically significant (*p* < 0.05) and were assessed for fit using the Hosmer–Lemeshow test. The predictive capacity of the models was evaluated using the area under the ROC (receiver operating characteristic) curve, and the area under the curve (AUC) values were used as an indicator of model performance. Cox–Snell and Nagelkerke R^2^ tests were employed to assess the proportion of variability in the dependent variables explained by the independent variables. Further technical details regarding the statistical methods and models are provided in Supplementary Materials File S1.

### 2.3. Qualitative Method

The research followed an interpretivist paradigm, adopting a case study design. The phenomenon under study refers to the perception of telemedicine adoption by workers in a specific context: a private healthcare network in Angola.

#### 2.3.1. Study Population and Sample

The target population for the study included mental health professionals within the private healthcare network. Non-probabilistic sampling was employed, with participants intentionally selected based on critical cases and the following criteria: being a physician or specialist in psychiatry or psychology; being an employee within the network in Luanda, male or female, regardless of years of experience in mental health; willingness and interest to participate in the study; and prior experience with telemedicine or lack thereof.

Participants were recruited via telephone by one of the researchers, who invited them to participate in the study and scheduled interviews with mental health specialists who voluntarily agreed to take part. A total of nine mental health professionals within the network were invited, of whom six initially agreed to participate, including three psychologists and three psychiatrists. Of these, five interviews were successfully conducted: three psychologists based in Luanda, working at the main facility and other mental health departments, representing 50% of psychologists employed within the network, and two psychiatrists based at the main facility, representing 66.6% of psychiatrists. One interview was canceled due to a language barrier, reducing the final number of valid interviews to five.

#### 2.3.2. Data Collection

Descriptive data collection was conducted using interactive techniques, including structured interviews scheduled according to the availability of psychologists and psychiatrists. Interviews were conducted by telephone for convenience and ease of recording, with participants’ authorization. Each interview was initially estimated to last one hour.

The data collection instrument was a structured questionnaire with open-ended questions prepared by the researchers. Although less flexible and limited to the questionnaire’s items, the structured questionnaire was deemed appropriate for the investigation context and mental health professionals, offering advantages such as standardized questions for all participants, efficiency, consistency, and easier comparison of responses.

The questions included in the interview guide were generally based on the pillars of the Technology Acceptance Model (TAM) [56,60]. TAM is a popular theory for assessing and predicting telemedicine acceptance and usage intention. While often used in quantitative components to study perceptions and contributing factors for technology acceptance, its structure was adapted to guide questionnaire development. Accordingly, TAM groups questions into the following: Perceived Usefulness of Telemedicine, Perceived Ease of Use, and Intention to Use, totaling 15 questions (see Supplementary Materials File S2).

#### 2.3.3. Analysis and Processing of Qualitative Data

Primary data analysis was conducted using thematic analysis, following the guidelines of Braun and Clarke [61]. Interview data were transcribed verbatim, with one hour allocated for every ten minutes of recording. Following transcription and participant anonymization, a pre-analysis phase began, involving data familiarization through repeated reading and note-taking with keywords for coding.

In the coding phase, codes were captured based on semantic meaning with potential for latent analytical significance from participants’ excerpts. Codes were assigned according to the predetermined organization of questions and question groups for deductive analysis, as well as based on interesting units identified in the text for potential inductive analysis.

Subsequently, themes and subthemes were defined for the inference phase, refined to ensure internal homogeneity and external heterogeneity. Analysis and result reporting involved data segment extraction and conceptual mapping. All thematic analysis steps were performed using MAXQDA (Version 2022, VERBI Software, Germany). qualitative analysis software. The data were triangulated with quantitative data to enable cross-verification of findings, confirm the accuracy and consistency of the results in both methods, and strengthen the overall validity and trustworthiness of the research.

Regarding reflexivity during data collection and analysis, reflective notes were maintained throughout the study to document and analyze thoughts, emotions, and potential biases arising during interviews and data analysis, aiming to recognize and mitigate researchers’ subjectivity.

### 2.4. Ethical Considerations

To conduct the study within the healthcare network, a request was submitted to the chairman of the management board and the Ethics Committee (approval code: 321413), which issued favorable opinions for the study, as well as to the management of partner units.

Participation in this study was voluntary and anonymous. Survey participants provided prior consent through the Google Forms questionnaire, acknowledging that the collected data would be used exclusively for scientific purposes. Similarly, interview participants provided written consent to participate in the study in accordance with the Declaration of Helsinki, and verbally consented to interview recording, knowing the recordings would be transcribed for data analysis. Participants’ confidentiality was maintained by collecting only general demographic data without specific identifiers in the questionnaires. Additionally, the interview data were transcribed with all participants anonymized.

## 3. Results

### 3.1. Quantitative Results

#### 3.1.1. Survey Participants’ Characteristics

From the sampling universe across three regions of Angola, including ten units within the private healthcare network and an estimated 2721 workers, the participation of n = 280 (10%) workers was obtained during February and March 2024. Of these, 0.7% (n = 2) declined to participate, and 1.1% (n = 3) were excluded due to having less than three months of experience within the network, resulting in a final sample of n = 275 workers.

The sample included a slightly higher participation of men (57.1%) than women (42.9%). The 25–34-year age group was the most represented, accounting for 52.0% (n = 143). Although the professional categories were relatively balanced, physicians stood out slightly, representing 28.7% (n = 79). Additionally, 73.8% of participants (n = 203) had more than three years of experience within the network (Appendix A, Table A1 Participant Characterization).

#### 3.1.2. Factors Influencing Telemedicine Adoption

The findings below highlight key factors influencing healthcare professionals’ predisposition to adopt telemedicine within the network, aligning with the study’s research questions.

In general, the collected sample highlighted that 83% of participants had never sought mental health professional help in person, while 85.5% reported mental health impacts due to work. Furthermore, 77.8% of healthcare workers within the network agreed that videoconferencing is a useful tool for mental health support.

Regarding videoconferencing, 82.9% of workers had previously used videoconference services, and 95.6% owned suitable devices for conducting them. Although 17.7% did not agree that their internet connection was strong enough for videoconferencing, most professionals preferred smartphones (47.3%) and computers (29.8%) as devices for conducting video consultations.

In terms of psychological or psychiatric support, 89.1% of workers stated they could attend a first video consultation, 88.0% could discuss their problems, 94.9% believed they could receive help for a psychological issue during a video consultation, and 86.9% could accept a medication prescription via video consultation.

Concerning the doctor–patient relationship, 86.9% of workers would feel comfortable doing a mental health video consultation. However, 51.3% were neutral or disagreed that a psychologist or psychiatrist could provide the same quality of care as in-person consultations. Additionally, only 56.0% agreed that video consultations are secure and respect privacy and confidentiality. Furthermore, 53.1% believed video consultations would allow for better personal and professional organization compared to in-person consultations. The full results are provided in Appendix A (Table A2).

#### 3.1.3. Comparison of Factors Influencing Telemedicine Adoption Between Headquarters and Partner Units

This section compares telemedicine adoption factors between the main facility and the partner units, highlighting key differences in access, perceptions, and usage patterns.

Regarding health units, the main facility accounted for 40.7% of participants (n = 112), representing 6% of the total workforce (n = 1941), a smaller proportion compared to partner units, which accounted for 59.3% (n = 163), representing 21% of total workers in partner facilities (n = 780) (Appendix A, Table A1).

Bivariate and multivariate analyses revealed similar results across six variables with statistically significant associations within the professional group (Appendix A—Table A3 and Table A4).

Workers from the main facility were four times more likely to have conducted video consultations than those from partner units [Exp(B) = 4.776; 95% CI = 1.498–15.374; *p* = 0.008] (Q9). Additionally, workers were eight times more likely to have access to materials/devices for conducting video consultations at the main facility compared to partner units [Exp(B) = 8.364; 95% CI = 1.061–65.947; *p* = 0.044] (Q10).

In terms of the perceived ease of access to in-person mental health care, workers from the main facility were less likely to report such ease compared to those from partner units [Exp(B) = 0.449; 95% CI = 0.260–0.775; *p* = 0.004].

The perception of being able to speak spontaneously with a mental health professional during a video consultation was lower among workers from the main facility compared to partner units [Exp(B) = 0.446; 95% CI = 0.260–0.765; *p* = 0.003] (Q23). Regarding the perception of security and respect for privacy during video consultations, workers from the main facility reported lower perceptions compared to partner units [Exp(B) = 0.473; 95% CI = 0.284–0.790; *p* = 0.004] (Q26).

Regarding the preferred frequency for conducting video consultations, workers from the main facility were three times more likely to choose the category “Annually, with additional sessions as needed” compared to those from partner units [Exp(B) = 2.726; 95% CI = 1.073–6.923; *p* = 0.035] (Q14, Appendix A Table A5). The validation results of the regression models presented significant values, with modest predictive capacity for variability in dependent variables (Appendix A Table A6).

#### 3.1.4. Complementary Results

This section presents additional statistically significant findings related to gender, age, and professional category, which influence perceptions and the adoption of telemedicine.

Complementary results revealed other statistically significant findings relevant to the research (Appendix A Table A7). Women (93.2%) were three times more likely than men (79.6%) to report that their mental health was impacted by work [Exp(B) = 3.522; 95% CI = 1.557–7.970; *p* = 0.002] (Q15). Gender was significantly associated with the preference for video consultation frequency (*p* = 0.020), where women were twice as likely to choose the category “As requested by workers at any time of the year” [Exp(B) = 2.288; 95% CI = 1.140–4.953] (Q14).

Among age extremes, workers aged 18–34 years (91.6%) were 11 times more likely than those aged 50 years or older (50.0%) to report they could discuss their problems during a video consultation [Exp(B) = 11.032; 95% CI = 3.094–39.335; *p* < 0.001] (Q17). Similarly, workers aged 18–34 years (91.0%) reported they could discuss themselves during a video consultation seven times more likely than workers aged 50 years or older (58.3%) [Exp(B) = 7.841; 95% CI = 2.071–27.025; *p* = 0.002] (Q19).

Administrative professionals (94.4%) were three times more likely to feel comfortable conducting a mental health video consultation compared to clinical professionals (84.2%) [Exp(B) = 3.180; 95% CI = 1.083–9.337; *p* = 0.035] (Q8). Furthermore, administrative professionals (84.7%) were also more likely to feel comfortable with the technology for conducting video consultations than clinical professionals (66.0%) [Exp(B) = 2.859; 95% CI = 1.412–5.790; *p* = 0.004] (Q27).

### 3.2. Qualitative Results

#### 3.2.1. Interview Participants’ Characteristics

From the interviewed mental health professionals (n = 5) who agreed to participate in this study, three were psychologists and two were psychiatrists, with an average age of 42.1 years and an average of 23 years of work experience within the healthcare network (Appendix B, Table A8). The psychologists represented 42.8% of the seven psychologists in the network, while the psychiatrists accounted for 66.6% of the three psychiatrists in the network.

#### 3.2.2. Themes from Qualitative Study

The deductive qualitative analysis identified three themes based on the predetermined structure of the interview, related to the perception, ease, and intention of using telemedicine in the form of video consultations. An additional relevant theme emerged outside the interview guide questions, related to perceptions of mental health in Angola as a barrier to telemedicine adoption.

#### 3.2.3. Theme 1 (Qualitative Data): Perceptions of Psychologists and Psychiatrists About Telemedicine

This theme presents the perspectives of mental health professionals regarding the adoption of telemedicine. It explores its perceived advantages for both providers and workers, as well as the technical and clinical challenges involved. These insights contribute to understanding telemedicine’s role in mental healthcare and align with the study’s objectives by identifying the key factors influencing its implementation.

Video consultations were viewed as a practice that could be adapted and structured within the private healthcare network, seen as innovative and beneficial. One interviewee emphasized that the confidential nature of video consultations could enhance adherence and ensure privacy. Among the five interviewees, four already had private experience with video consultations, frequently conducting them with patients from Portuguese-speaking African countries (PALOP), both within and outside Luanda.

“(…) Three times a week, and I’ve been doing this for more than three years. Ah, since COVID, since the COVID pandemic started, I began doing video consultations. Since COVID, I have patients here, I have patients abroad, I have patients in the provinces, so I do this—it’s a routine medical practice.”

Interviewee 3, Pos. 24

Professionals identified several advantages of using video consultations for themselves as providers, including flexibility to consult both at healthcare facilities and at home. The possibility of remote interventions, extended consultation periods, optimized idle time, time management, and the ability to record consultations for later analysis were highlighted as positive aspects.

The interviewees considered that video consultations reduce workers’ exposure, potentially eliminating the fear of attending consultations. Additionally, cost reduction, particularly associated with travel, was noted as an important benefit.

Another identified aspect was the potential to expand care coverage within the healthcare network, promoting self-management of mental health and offering the choice between in-person and telemedicine formats, especially for workers outside the country’s capital (Luanda).

Challenges associated with video consultations included several aspects of technological infrastructure. All interviewees highlighted internet quality as a difficulty that could limit consultation effectiveness. Additionally, the need to protect clinical and administrative data to ensure confidentiality was mentioned.

“Regarding the means as well, the quality of the internet, sometimes the person is speaking, the internet freezes, then suddenly it comes back, and during the time it comes back, the person is saying, ‘Hello, doctor, hello, hello, hello’, and I’m also saying, ’So-and-so, so-and-so, are you there?”

Interviewee 5, Pos. 40

Other challenges included the quality and access to electronic devices, and interruptions in the patient’s environment. Moreover, a lack of familiarity with technology among some users and the need to raise awareness about video consultations were noted as significant obstacles.

Providers perceived video consultations as more demanding than in-person consultations, requiring greater clarity, attention, and the ability to engage the patient. Limitations in observing patients’ non-verbal language were noted. However, it was considered that the dialogic nature of mental health specialties makes clinical assessment more feasible.

“The mental health consultation is not like an orthopedic consultation, or like a surgical consultation. I don’t necessarily need to touch the patient, I need to talk, to see, to see their facial expression, face, and voice. Nothing more. And at a distance, I do my work.”

Interviewee 3, Pos. 44

#### 3.2.4. Theme 2 (Qualitative Data): Perceptions of Telemedicine Ease of Use

This theme examines professionals’ views on the usability of telemedicine tools, analyzing how the ease of use influences their willingness to adopt the technology. It also explores device preferences, prior experiences, and training needs, providing a comprehensive understanding of how usability impacts telemedicine adoption.

Interviewees did not find video consultations difficult, given their familiarity with videoconferencing tools. They showed a clear openness and willingness to learn new platforms and acquire new skills.

“(…) I am willing to learn other apps, my mind is still open to learning.”

Interviewee 1, Pos. 71

The computer was considered the preferred device, offering greater comfort, enabling observation of the patient’s environment, and avoiding distractions common with mobile phones, making consultations more focused and effective. However, one interviewee also considered mobile phone use as valid. Professionals recognized the importance of quick training sessions for themselves and staff, focusing on familiarization with software and clarifying the need and advantages of video consultations. However, one interviewee deemed training on software use unnecessary but considered it crucial to clarify the prerequisites for video consultations to align expectations and processes.

“As I said, the app or platform has to be as user-friendly as possible. It has to be less, let’s say, complicated, so that with 15 or 30 min of information, someone with the ability to handle an electronic device can enter, exit, input data, and then access the video and interact with the patient, right?”

Interviewee 2, Pos. 116

#### 3.2.5. Theme 3 (Qualitative Data): Intention to Use Telemedicine

This theme explores the determinants influencing professionals’ intention to integrate telemedicine into their practice. It examines factors such as institutional support, financial policies, and the key actions professionals perceive as essential for the successful implementation and use of telemedicine.

Providers demonstrated a clear intention to adopt telemedicine in the form of video consultations. They perceived that integrating telemedicine into the mental health department’s activities would result in greater efficiency without requiring the creation of a new service. This efficiency would also depend on robust organization of care processes and effective agenda management.

“I don’t think there needs to be an independent service. I believe that, for efficiency, what should be considered is integrating this into the overall activities of the mental health department.”

Interviewee 2, Pos. 135

Mental health support via telemedicine was viewed as a way to enhance client satisfaction, improve employee health, and increase efficiency by reducing time spent on administrative tasks. Interviewees concluded that the doctor–patient relationship would not be compromised in video consultations, emphasizing the importance of confidentiality to ensure workers’ adherence. Interactions should adhere to legal, ethical, and deontological standards to ensure security and privacy.

Regarding factors influencing professionals’ decisions to adopt video consultations, interviewees demonstrated a high readiness to provide care and assist patients without access to mental health services. Other influencing factors included the possibility of consulting from home for convenience and extending work schedules.

Financial motivation, linked to increased patient numbers, was considered a factor influencing video consultation adoption, along with clear payment policies and good financial management. One professional emphasized that payment should be based on the time allocated and that the risk of cancellation should be borne by patients for efficient cancellation management.

“A good administration of this service, fees, that’s important. I will be paid for my time available for these online consultations. If the patient misses the appointment, it’s no longer my problem.”

Interviewee 1, Pos. 125

Implementing telemedicine was perceived as beneficial for supporting mental health within the network, addressing unmet needs, and enabling potential expansion to other regions or networks. Many professionals believed video consultations should have been implemented already. Professionals highlighted several actions necessary for integrating video consultations into the network, such as demonstrating the losses from not integrating telemedicine to support workers’ mental health, preparing and educating workers on its importance, and formalizing a robust proposal for the management board.

“(…) And then the network needs to invest, because everything that comes next is investment. Electronic equipment is investment, maintenance of all equipment, system, and internet are investments. So, the biggest highlight for me would be the investment.”

Interviewee 5, Pos. 112

Additionally, other actions explored by professionals for implementing video consultations included creating a user-friendly platform, promoting and publicizing the service, training data managers, and efficient service management. Investment in technological infrastructure was seen as a priority to ensure the suitability of video consultation practices. The ideal frequency for video consultations was perceived as multifactorial, depending on various factors such as specialist availability, workload distribution, patient demand, and clinical conditions.

#### 3.2.6. Theme 4 (Qualitative Data): Perceptions of Mental Health in Angola

This theme discusses the broader context of mental health in Angola, considering sociocultural factors that influence access to care and the adoption of telemedicine. It highlights challenges such as stigma and healthcare infrastructure limitations while emphasizing the potential of telemedicine as a solution to improve mental health services.

General perceptions of mental health in Angola may influence telemedicine adoption. Professionals noted that there is no culture of seeking help, with mental health being poorly recognized by the general population, which could act as a barrier to adopting video consultations for mental health support.

In the hospital context, mental health was perceived by interviewees as an element directly influencing workers’ functionality. Professionals emphasized that a hospital worker without mental health would be unable to provide adequate care, potentially resulting in medical errors or presenteeism, compromising care quality highlighting the need for mental health support for professionals.

“Employees will only function if they have mental health, because mental disorders compromise functionality at work. (…) There can be high losses, from misuse of materials, work accidents involving the patient, even leading to the patient’s death.”

Interviewee 5, Pos. 126

However, another interviewee noted that beyond the lack of recognition of mental health needs, fear of criticism prevents professionals from seeking mental health care. This perception could affect adherence to video consultations.

“When working in a hospital unit, it’s very visible that people are afraid to go for a consultation, mainly due to criticism from colleagues.”

Interviewee 1, Pos. 171

Video consultations could impact workers’ productivity. One professional suggested that a mentally healthy worker is more productive, and failing to invest in workers’ mental health could lead to potential institutional losses. All relevant interview segments are provided in Supplementary Materials File S2.

“A mentally healthy worker can easily get motivated, and when well-motivated, they produce a lot. (…) The company is always ahead of problems, not behind. So, the money the company doesn’t invest more in mental health prevention, it’s actually losing.”

Interviewee 5, Pos. 135

### 3.3. Integration of the Results

The comparative analysis of survey and interview data integrated the findings, ensuring cross-verification, rigor, and consistency in identifying the factors influencing telemedicine adoption and healthcare providers’ perception of telemedicine as a tool for mental health support.

Both qualitative and quantitative methods indicate a low help-seeking culture for mental health among workers. While employees perceive limited access to in-person consultations, healthcare providers emphasize that video consultations can improve access to mental health care. Regarding technological infrastructure challenges, internet connection quality emerges as a crucial barrier. However, participants are familiar with videoconferencing tools, and video consultations are perceived positively in both surveys and interviews. Privacy and confidentiality concerns were noted by workers during video consultations, whereas providers not only recognized these concerns, but also highlighted that teleconsultations may reduce exposure and offer greater discretion. Additionally, most participants expressed a high willingness to engage in video consultations, and healthcare professionals emphasized the need for a validation mechanism for electronic prescriptions (Table 1).

## 4. Discussion

### 4.1. Discussion of Quantitative Results

In this study, the main factors influencing the predisposition to adopt telemedicine as a tool to support mental health among workers in the private healthcare network are workers’ mental health status, previous consultation experiences, perceptions of access to in-person consultations, perceptions of video consultations, and the doctor–patient relationship. These factors are influenced by worker gender, healthcare unit, age group, and professional category.

Most workers had never sought help from a mental health professional in person, despite reporting work-related mental health impacts. The literature identifies various barriers to seeking mental health care, such as fear of discrimination, perceived lack of need for mental health care, confidentiality concerns, and potential career impacts [62,63,64].

Workers at the main facility reported less ease of access to in-person mental health consultations compared to those from partner units, potentially due to the current requirement for preliminary medical evaluation. This highlights the need for a specific mental health support program within the network.

In this study, women were more likely to report mental health impacts compared to men. This finding aligns with the literature, indicating greater mental health impacts among female healthcare professionals during the pandemic [5,15,65]. Although this study did not assess the mental health status or its impacts among professionals, this information is critical for tailoring interventions within the healthcare network, given women’s potentially differing needs.

Younger workers (18–34 years) were more likely to discuss themselves during a video consultation compared to those aged 50 years or older, suggesting that younger individuals are more comfortable with technology. Strategies to engage older workers should be developed.

Overall, workers had positive perceptions of mental health support via video consultations. They believed they could discuss their problems, receive help for psychological issues, accept a medication prescription, and have a first video consultation. These findings demonstrate professionals’ openness to conducting mental health video consultations. However, half of the professionals were neutral or disagreed that psychologists or psychiatrists could provide the same quality of care through video consultations as in-person consultations, indicating concerns about the efficacy of this care format.

Although professionals perceived that conducting mental health video consultations could be comfortable, concerns regarding security, privacy, and confidentiality remained. Similar concerns were reflected in the study by Cormi et al. (2021) [66], conducted in a different geographical and organizational context.

The findings of this study suggest that a telemedicine implementation model must consider the factors influencing adoption and significant differences between the main facility and partner units. It is essential to sensitize workers and demonstrate the importance of video consultations.

Administrative staff, compared to clinical staff, were more likely to feel comfortable conducting mental health video consultations if experiencing psychological issues and felt more at ease with technology. Cormi et al. (2021) [66] identified that clinical staff perceived remote support less favorably, aligning with this study’s findings. These results suggest that administrative workers within the network may be more receptive to remote mental health support, which highlights the need to involve clinical professionals.

Video consultations in mental health should meet technological standards outlined by the World Psychiatric Association (WPA) [67]. Challenges such as inadequate home internet and limited access to devices, particularly in partner units, were identified. Mitigation strategies include pre-assessing home conditions, using audio calls during interruptions, or equipping health units with dedicated teleconsultation rooms [67].

The preference for mobile phones as the primary device for video consultations was notable and aligns with a study conducted in a private institution in Kenya [68], likely due to the accessibility and portability of these devices. Workers also expressed a preference for conducting video consultations on an annual basis, with additional sessions as needed. These preferences indicate a demand for structured and flexible telemedicine programs tailored to individual needs and organizational contexts.

### 4.2. Discussion of the Qualitative Results

This study aimed to explore the perceptions of mental health professionals regarding telemedicine and its adoption as a support tool for the mental health of workers within a private healthcare network. The findings revealed that several factors influence the adoption of telemedicine.

#### 4.2.1. Theme 1: Perceptions of Psychologists and Psychiatrists on the Use of Telemedicine

The advantages of telemedicine identified in this study align with the literature, particularly in improving access to care for individuals facing mobility limitations [69]. Telemedicine has also been shown to enhance patient engagement, satisfaction, and adherence, while promoting continuity in the therapeutic process [70,71]. Additionally, it offers greater comfort and flexibility compared to in-person consultations [72,73].

Professionals in this study viewed telemedicine as a complementary tool rather than a replacement for in-person care. This perspective supports a hybrid care model, as highlighted by Moeller et al. (2022) [71] and Uscher-Pines et al. (2022) [70], where video consultations expand care options and improve overall quality. However, prior experience with telemedicine appears to influence perceptions, as evidenced by Hoffmann et al. (2020) [74], where professionals with no prior experience viewed telemedicine as suitable only for initial consultations or diagnostics.

Confidentiality and privacy were identified as key advantages of telemedicine, particularly in reducing exposure to coworkers. However, professionals acknowledged challenges related to maintaining privacy in home environments, an issue also noted in other studies [68,73,75]. These findings highlight the need to inform employees of the basic principles for conducting consultations to minimize these challenges and need for designated teleconsultation rooms in healthcare facilities to ensure safety and comfort, as recommended by the World Psychiatric Association (WPA) [67,76].

Telemedicine was also perceived as effective in supporting mental health self-management, enabling continuity in care without compromising the doctor–patient relationship. These findings align with studies from South Africa, where telemedicine facilitated both brief interventions and longer therapeutic sessions [75]. Additionally, telemedicine was noted to increase patients’ comfort in discussing sensitive topics [77], and studies suggest that therapeutic relationships can be maintained effectively in virtual formats [78].

Regarding the challenges, one of the most central issues was the reduced ability to observe patients’ non-verbal language, a concern consistent with the literature [68,69,73,74,77,79]. However, “empathic accuracy” (the ability to perceive how the patient feels) and the “therapeutic alliance” (the establishment of a trustworthy and collaborative relationship) are not significantly affected in the telepsychiatry format [78], which justifies the development of strategies to focus on verbal language during the therapeutic process to establish a satisfactory doctor-patient relationship.

Professionals also highlighted the increased mental effort required during video consultations, consistent with findings from Goldschmidt et al. (2021) [75] and Buckman et al. (2021) [72]. The telemedicine model, while convenient, can be more exhausting than in-person care, underscoring the need for adequate workload management.

The technical challenges identified in this study, including the need for secure, user-friendly platforms and data protection, align with findings from other studies [69,71,73]. The WPA [67,80] emphasizes prerequisites such as interoperability, compatibility, patient safety, and data security, which must be addressed to ensure successful telemedicine implementation within the network.

Internet quality was identified as a challenge for delivering care via video consultations, especially in peripheral regions of Angola. This challenge was also identified in Kenya [68], and South Africa, where access and costs hindered internet use, particularly in underserved populations [75]. Even in London, the quality of the internet connection and the lack of suitable devices were barriers highlighted by Buckman et al. 2021 [72], despite being a completely different context. Utilizing available resources and adapting specific rooms in partner units could mitigate challenges faced by workers, such as connectivity issues or inadequate devices.

#### 4.2.2. Theme 2: Perceptions of the Ease of Using Telemedicine

Professionals perceived video conferencing tools as easy to use, which positively influenced their high intention to use them. In the study by Knott et al. 2020 [76], professionals who considered video conferencing technologies difficult demonstrated a greater tendency to highlight limitations rather than benefits, adopting a more resistant attitude toward telemedicine compared to those who found the tools easy to use. This finding reinforces the conclusion that the perception of the ease of video consultations can influence professionals’ adoption.

The computer was the preferred device for most professionals due to its broader visual coverage and lower risk of interruptions from notifications, unlike mobile phones. In contrast, in Kenya, mobile phones were preferred, possibly due to the experience with more user-friendly platforms [68]. Additionally, the ability to study the patient’s home environment, providing additional information, is an advantage identified in agreement with the literature [69,79,81].

Professionals emphasized the importance of training, both for themselves and for employees receiving care via telemedicine. Training is widely supported in the literature as essential for developing technical competence and ensuring the effective use of telemedicine technologies [80]. A lack of exposure to telemedicine during undergraduate education may create barriers, as noted by Knott et al. (2020) [76]. However, well-designed training programs can address these challenges, shaping positive attitudes toward telemedicine adoption.

In the literature, the perceptions of training activities are explored as opportunities for professionals to familiarize themselves with video consultations [74], as well as to optimize care through training focused on resolving issues that may arise during sessions, video tutorials to learn the functions of video platforms, and practical usage simulations [72]. In the study by Lipschitz et al. (2022) [73], professionals identified the need to receive a best practices manual that would provide technical, legal, and ethical standards for using telemedicine. Conversely, in the study by Moeller et al. (2022) [71], it was concluded that in scenarios of a high workload, training may be seen as a burden and negatively influence the perception of telemedicine adoption. These findings reinforce the importance of training activities in telemedicine for both providers and employees within the healthcare network.

Training opportunities, as highlighted in the literature, should include tutorials for platform functionality, simulations for practical application, and troubleshooting strategies [72,74]. Lipschitz et al. (2022) [73] noted the value of best practice manuals, outlining the technical, legal, and ethical standards. However, Moeller et al. (2022) [71] emphasized that in scenarios of high workload, training may be perceived as a burden, potentially negatively influencing professionals’ attitudes toward telemedicine adoption. These findings reinforce the importance of tailoring training programs to the specific needs of healthcare professionals, ensuring that both providers and employees can fully benefit from telemedicine.

#### 4.2.3. Theme 3: Intention to Use Telemedicine

All professionals in this study demonstrated a strong intention to conduct video consultations within the healthcare network, with key factors influencing this decision, such as the feasibility of the project. Uscher-Pines et al. (2020) [69] similarly observed that while professionals utilized video consultations during the pandemic, many preferred returning to in-person care due to uncertainties about the long-term viability of telemedicine. This finding underscores the importance of institutional strategies to ensure the sustainability of telemedicine, which is critical for professional adherence.

Financial policies were another significant factor influencing adoption. In the United States, unclear finance policies created perceptions that hindered the continuity of telemedicine [69]. Similarly, Lipschitz et al. (2022) [73] identified funding barriers imposed by insurers as a challenge. In Denmark, professionals received financial incentives to encourage the use of video consultations, but these alone were insufficient for long-term adoption [71]. Some professionals in this study suggested that payment should still apply if patients fail to attend consultations, a view echoed in a German study [74], highlighting the importance of financial incentives for adoption and continuity.

Institutional support also plays a crucial role in professionals’ willingness to adopt telemedicine. Effective service management, including hospital and Information and Communication Technology (ICT) support, along with immediate technical assistance during video consultations, significantly impacts adoption, as highlighted in previous studies [71,79].

Professionals perceive the ideal frequency of video consultations as multifactorial, guided by the patient’s clinical condition, with a higher frequency in psychology than in psychiatry. In the literature, frequency is also viewed as multifactorial, considering that not all clinical cases are suitable for telemedicine. However, removing access barriers generally results in more frequent and patient-centered sessions [70,71,73,79].

Professionals emphasized that video consultations must uphold privacy, ethical, and deontological standards, supported by informed consent. This aligns with the “Telepsychiatry Global Guidelines”, which underline the importance of privacy, confidentiality, and ethical compliance in telepsychiatry [67,80].

#### 4.2.4. Theme 4: Perceptions of Mental Health in Angola

In Angola, the social constructs surrounding mental health represent, in part, a significant barrier to accessing mental health care, which can negatively impact the adoption of video consultations. In the context of telepsychiatry, it is essential that mental health professionals provide culturally competent care [67], tailored to the country’s cultural diversity.

In the present study, it was identified that fear of criticism from colleagues is a factor that discourages employees from seeking mental health care. Although there are circuits in place at the main facility, it is necessary to expand coverage to partner units. Telemedicine was considered an effective solution in this context.

Respondents indicated that mental disorders directly affect the productivity of healthcare professionals, leading to presenteeism, medical errors, and financial losses for the Angolan healthcare network. Despite the barriers to adopting telemedicine, they highlighted essential actions for its implementation in supporting mental health and expressed an optimistic perspective on the benefits and the crucial role of telemedicine in the future.

Raising workers’ awareness about the importance of self-management of their mental health and its implications across various domains of their lives is a crucial step in managing networks most valuable asset: its human capital.

#### 4.2.5. Study Limitations

This study has several limitations. First, its observational, cross-sectional, and descriptive nature does not allow for causality inference. Additionally, its external validity is limited since results were based on the private healthcare context in Angola and may not generalize to public or international healthcare settings. While offering valuable insights for low- to middle-income countries, findings should be cautiously compared.

Another limitation concerns the consideration of costs as a factor influencing the adoption of telemedicine. In the network, the costs associated with workers’ healthcare are subsidized, with a relatively low percentage. This aspect was not the subject of study.

The topic of telemedicine as a support tool for the mental health of healthcare professionals represents a gap in the literature, with no previous studies available, which limits the discussion of the results within a broader body of the literature.

Structured questionnaires can limit data collection, as their standardized and pre-determined nature restricts the exploration of unexpected insights. Moreover, this study focused solely on telemedicine via video consultations, without analyzing the use of audio calls. Another limitation was the small number of interviewed professionals, which reflects the overall shortage of mental health professionals in the private network. While data saturation was not achieved, the insights gathered from the five participants provided valuable contributions to understanding the phenomenon under study. Despite this, the sample represented a significant proportion of the network’s psychologists (42.8%) and psychiatrists (66.6%), ensuring diverse perspectives within the study’s scope.

#### 4.2.6. Telemedicine-Based Mental Health Model and Operational Circuit

**Figure 1:**
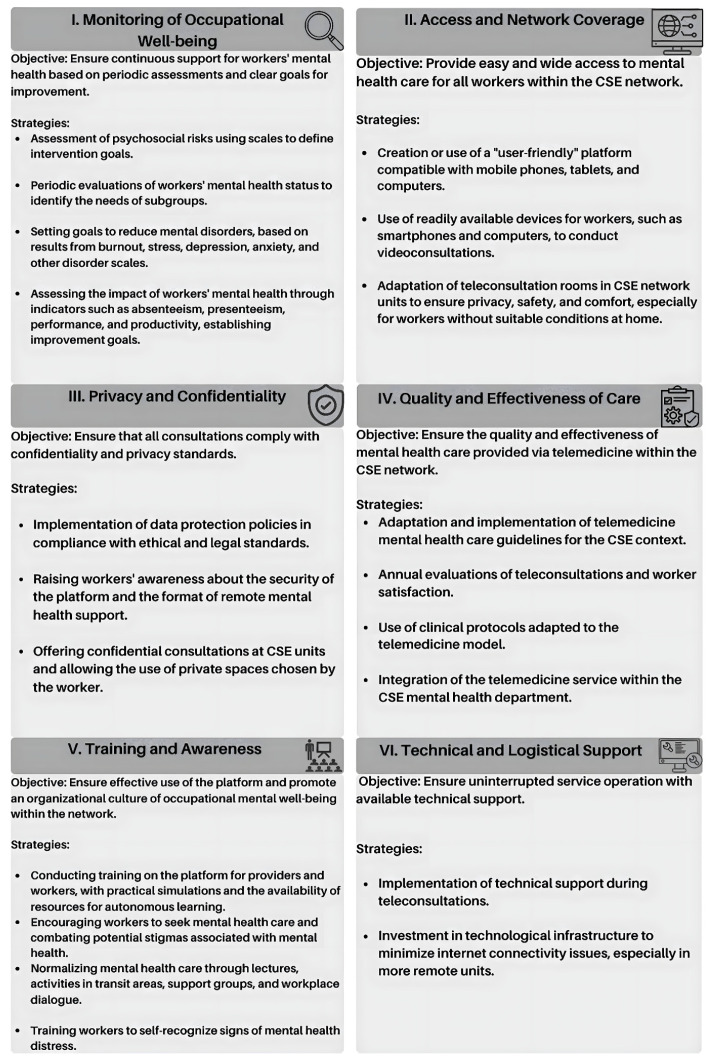
Pillars of the mental health model via telemedicine.

The development of the telemedicine model for mental health support (Figure 1) is grounded in both the empirical findings of the study and established evidence in the literature. The first pillar, “Occupational Well-Being Monitoring”, is justified by the high value workers place on regular mental health assessments. The use of validated scales to evaluate psychosocial risks and measure impacts such as burnout, stress, depression, and anxiety facilitates the establishment of targeted intervention goals for specific subgroups. Instruments like the Quality of Work Life Questionnaire, Job Content Questionnaire, and the Copenhagen Psychosocial Questionnaire, among others, provide a robust framework for monitoring indicators such as absenteeism, presenteeism, and productivity [82,83,84,85,86,87,88,89,90].

The second pillar, “Access and Network Coverage”, was formulated in response to the identified barriers to mental health care access, particularly in partner units. Creating a user-friendly platform that is compatible with smartphones, tablets, and computers is intended to overcome geographical and technological limitations, ensuring that all workers can readily access telemedicine services.

The third pillar, “Privacy and Confidentiality”, addresses workers’ frequent concerns regarding the security of video consultations. By implementing stringent data protection policies in line with ethical and legal standards, and by offering secure environments—either within the facility or in private settings chosen by the worker—this pillar aims to build trust and encourage the adoption of telemedicine.

The fourth pillar, “Quality and Efficacy of Care”, responds to uncertainties about the effectiveness of mental health services delivered via telemedicine. Continuous adaptation of international guidelines to the specific context of the network, coupled with the integration of tailored clinical protocols and periodic evaluations of teleconsultations and worker satisfaction, ensures that the service maintains high standards of quality and safety.

The fifth pillar, “Training and Awareness”, emphasizes the critical importance of equipping both providers and workers with the necessary skills for the effective use of videoconferencing platforms. Practical training sessions, simulations, and the availability of self-learning resources foster technical competence, while initiatives aimed at reducing stigma and promoting mental health awareness contribute to a supportive organizational culture. Finally, the sixth pillar, “Technical and Logistical Support”, directly addresses infrastructural challenges such as connectivity issues and inadequate access to technology. Robust technical support and targeted investment in technological infrastructure are essential for ensuring the uninterrupted, efficient delivery of video consultations.

**Figure 2:**
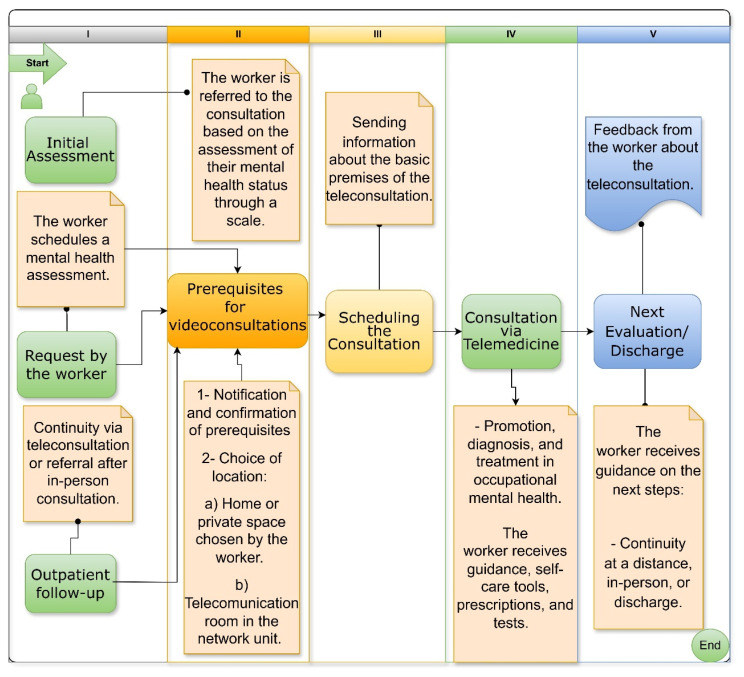
Operational circuit of the mental health model via telemedicine.

The operational circuit (Figure 2) was derived by integrating empirical evidence from our study with established telepsychiatry guidelines, thereby ensuring that each process reflects both research findings and best practices. For instance, the initial assessment phase, which utilizes standardized scales, emerged from data underscoring the importance of continuous mental health monitoring, a key aspect of occupational well-being. This step also accommodates alternative entry points into care, acknowledging that some workers may follow more traditional pathways.

Similarly, the requirement to verify technical and environmental prerequisites for videoconferencing was shaped by both our study’s insights and international recommendations [67,76], emphasizing the need for secure, private, and technologically adequate settings.

The design of the scheduling phase, which includes the dissemination of fundamental telemedicine information, was informed by evidence showing that clear communication enhances worker preparedness and reduces uncertainties about the process. Furthermore, the consultation stage was structured to combine clinical care with supportive measures—such as self-care guidance and appropriate referrals—reflecting findings that a holistic approach improves treatment outcomes. Lastly, the follow-up phase, where future care is planned and feedback is systematically collected, was justified by global telepsychiatry standards that advocate for continuous quality improvement.

## 5. Conclusions

This study demonstrated a positive perception of telemedicine as a tool for supporting mental health within the private healthcare network. However, its implementation must be culturally adapted, addressing specific needs such as the greater mental health impact reported by female healthcare professionals, the inclusion of older and clinical workers in telemedicine use, and differences across healthcare units.

The adoption of telemedicine is influenced by technological challenges, including internet quality, access to appropriate equipment, and the need for user-friendly and secure platforms. Privacy, confidentiality, and data protection concerns were critical factors for workers, highlighting the necessity of clear institutional policies in these areas. For providers, key factors influencing telemedicine adoption include ease of technology use, project feasibility, management support, payment policies, and compliance with legal, ethical, and professional standards. These factors have direct implications for hospital administration in designing effective occupational mental health interventions using telemedicine. Strengthening professionals’ mental well-being can yield institutional and healthcare benefits, including increased productivity, job satisfaction, care quality, and reduced absenteeism, presenteeism, turnover, and medical errors.

Additionally, this study identified the need to raise awareness among healthcare professionals to foster an organizational culture that normalizes and prioritizes mental health. This study makes a significant contribution to the literature, as the first in Angola to explore occupational mental health among healthcare professionals and digital interventions. Future research should assess psychosocial risks among network professionals, analyze their actual mental health status, and identify the most prevalent mental disorders and their key predictors, such as gender, age, years of service, healthcare unit, and professional category.

## Figures and Tables

**Table 1:** Integration of the results.

Survey Results	Interview Findings
83% of participants never sought in-person mental health support	Interviewees perceive a lack of a help-seeking culture in mental health.
77.8% of healthcare network workers agree that video consultations are a useful tool for mental health support	Interviewees highlighted the flexibility, cost reduction, lower exposure, and ease of use of video consultations.
82.9% of workers have used videoconferencing services	All interviewees have experience using videoconferencing services.
65.5% have a neutral or disagreeing opinion on the ease of access to in-person mental health consultations	Interviewees perceive that video consultations can improve access to mental health care.
17.7% do not agree that their internet connection is strong enough for video conferencing	Internet quality was identified by interviewees as a major challenge.
89.1% of workers would be willing to have an initial video consultation	All interviewees expressed a strong intention to engage in video consultations within the network.
56.0% agree that video consultations are secure and respect privacy; 94.5% have a space where they can speak freely during a video consultation	Interviewees noted that video consultations can reduce exposure but acknowledged potential interruptions in the patient’s environment.
A large proportion of professionals prefer smartphones (47.3%) for video consultations	Most interviewees preferred using a computer for video consultations.
86.9% of workers would accept prescription issuance during a video consultation	Professionals emphasized the need for a mechanism to validate prescribed prescriptions.

## Data Availability

The datasets generated during or analyzed during the current study are available upon reasonable request to the corresponding author.

## Supplementary Materials

The following supporting information can be downloaded at: https://www.mdpi.com/article/10.3390/ijerph22040565/s1, File S1 includes: (1) Cultural Adaptation of the Questionnaire and (2) Questionnaire—Telepsychiatry for All, while File S2 contains: (1) Interview Script and (2) Qualitative Results—Relevant Segments of the Interview.

## Appendix A

**Table A1:** Characterization of the participants.

Sample Characterization (n = 275)	Category	Count	% of Column N
Participant Gender	Male	157	57.1%
	Female	118	42.9%
	Subtotal	275	100%
Age Group	18–24 years	12	4.4%
	25–34 years	143	52.0%
	35–49 years	108	39.3%
	50–60 years	8	2.9%
	Over 60 years	4	1.5%
	Subtotal	275	100%
Participant Professional Category	Physician	79	28.7%
	Nurse	63	22.9%
	Technician	61	22.2%
	Receptionists and Administrative Staff	72	26.2%
	Subtotal	275	100%
Work Experience in the Network	Less than 1 year	15	5.5%
	1 to 3 years	57	20.7%
	Over 3 years	203	73.8%
	Subtotal	275	100%
Healthcare Unit	Main Facility	112	40.7%
	Partner units	163	59.3%
	Subtotal	275	100%

Source: database of the survey “Telepsychiatry for Everyone” Applied in a Healthcare Network in Angola, 2024.

**Table A2:** Factors influencing the adoption of telemedicine.

Survey Results: “Telepsychiatry For Everyone” Applied in a Healthcare Network in Angola, 2024	Category	Count	% of Column N
Q6. Have you ever sought help from a mental health professional for psychological or psychiatric reasons, in-person for yourself?	Yes	45	16.4%
	No	230	83.6%
Q7. Have you ever used services like Zoom, Skype, Teams, WhatsApp, Messenger, among others, for videoconferencing?	Yes	228	82.9%
	No	47	17.1%
Q8. If you had a psychological problem, would you feel comfortable doing a videoconference with a mental health professional?	Yes	239	86.9%
	No	36	13.1%
Q9. Have you ever had a videoconference with a health professional as the patient?	Yes	16	5.8%
	No	259	94.2%
Q10. Do you have a device that allows you to conduct a videoconference (smartphone, computer, tablet, etc.)?	Yes	263	95.6%
	No	12	4.4%
Q11. Is your internet connection strong enough to conduct a videoconference (on your phone or at home)?	Yes	228	82.9%
	No	47	17.1%
Q12. Do you have a space where you can be calm and talk freely for at least 30 min during a videoconference?	Yes	260	94.5%
	No	15	5.5%
Q13. Which of the following devices would you preferably choose to conduct videoconferences? (Please check your preferred options)	Smartphone	130	47.3%
	Computer	82	29.8%
	Computer, Tablet	2	0.7%
	Tablet	19	6.9%
	Smartphone, Tablet	3	1.1%
	Smartphone, Computer	27	9.8%
	Smartphone, Computer, Tablet	12	4.4%
Q14. What frequency do you consider appropriate for psychology/psychiatry videoconferences at your unit? (Please select only one option)	Annually, during workers’ health check-ups	57	20.7%
	Annually, with additional sessions as needed	37	13.5%
	Quarterly	36	13.1%
	Semiannually	34	12.4%
	As requested by workers, at any time of the year	111	40.4%
Q15. Have you ever felt an impact on your mental health due to your work? (Including stress, insomnia, anxiety, burnout, etc.)	Yes	235	85.5%
	No	40	14.5%
Q16. If faced with a psychological problem, I think videoconferencing would be:	Easier than an in-person consultation	104	37.8%
	Equivalent to an in-person consultation	86	31.3%
	More difficult than an in-person consultation	52	18.9%
	I would not do a videoconference for a psychological problem	33	12.0%
Q17. During a videoconference, I could talk about my problems:	Yes, I could	242	88.0%
	No, I could not	33	12.0%
Q18. During a videoconference, I could do a first consultation:	Yes, I could	245	89.1%
	No, I could not	30	10.9%
Q19. During a videoconference, I could talk about myself:	Yes, I could	244	88.7%
	No, I could not	31	11.3%
Q20. During a videoconference, I could receive help for a psychological problem:	Yes, I could	261	94.9%
	No, I could not	14	5.1%
Q21. During a videoconference, I could accept a medication prescription:	Yes, I could	239	86.9%
	No, I could not	36	13.1%
Q22. I have easy access to in-person psychological and/or psychiatric care at the clinic: (Indicate your level of agreement with this statement)	Agree	95	34.5%
	Disagree/Neutral	180	65.5%
Q23. During a videoconference, I think I can spontaneously talk to my psychiatrist or psychologist about what worries me, even if they don’t ask:	Agree	186	67.6%
	Disagree/Neutral	89	32.4%
Q24. With videoconferencing, I think my psychiatrist or psychologist is capable of offering me the same quality of care they would offer in-person:	Agree	134	48.7%
	Disagree/Neutral	141	51.3%
Q25. During a videoconference, I think the doctor-patient relationship with a psychiatrist or psychologist would be satisfactory for me:	Agree	167	60.7%
	Disagree/Neutral	108	39.3%
Q26. Videoconferences are safe and respect my privacy and medical confidentiality:	Agree	154	56.0%
	Disagree/Neutral	121	44.0%
Q27. I feel sufficiently comfortable with the technology to do a videoconference:	Agree	195	70.9%
	Disagree/Neutral	80	29.1%
Q28. Considering my personal and professional limitations, a psychiatry or psychology videoconference would allow me to organize myself more easily compared to an in-person consultation:	Agree	146	53.1%
	Disagree/Neutral	129	46.9%
Q29. I think psychiatry videoconferencing is a useful tool for addressing workers’ mental health in my unit:	Agree	214	77.8%
	Disagree/Neutral	61	22.2%

**Table A3:** Bivariate analysis: Chi-square with risk estimate.

Question No.	Category	Relative Frequency (Yes)	X^2^ (Pearson’s Chi-Square)	df	*p*-Value	OR	95% CI
Q9							
	Main facility	10.7%	8.266	1	0.004	4.770	[1.497–15.199]
	Partner units	2.5%					
Q10							
	Main facility	99.1%	5.454	1	0.02	8.033	[1.022–63.132]
	Partner units	93.3%					
Q14							
	Main facility	23.2% (*)	18.291	4	0.001	N/A	N/A
	Partner units	6.7% (*)					
Q22							
	Main facility	26.8%	5.032	1	0.025	0.552	[0.327–0.930]
	Partner units	39.9%					
Q23							
	Main facility	58.9%	6.545	1	0.011	0.514	[0.308–0.859]
	Partner units	73.6%					
Q26							
	Main facility	47.3%	5.776	1	0.016	0.551	[0.339–0.898]
	Partner units	62.0%					

Q9—Have you ever had a videoconference with a healthcare professional as the patient? Q10—Do you have a device that allows you to conduct a videoconference (smartphone, computer, tablet)? Q14—What frequency do you consider appropriate for psychology/psychiatry videoconferences at your unit? (*) Relative frequency for the category “Annually, with additional sessions as needed”. Q22—I have easy access to in-person psychological and/or psychiatric care at the clinic. Q23—During a videoconference, I think I can spontaneously talk to my psychiatrist or psychologist about what worries me, even if they don’t ask. Q26—Videoconferences are safe and respect my privacy and medical confidentiality.

**Table A4:** Multivariate analysis: binary logistic regression.

Question No.	Category	Coefficient B	S.E	Wald	Sig	Exp(B)	95% CI (Exp(B))
Q9							
	Main facility	1.156	0.59	6.982	0.008	4.776	[1.498–15.374]
	Constant	−3.670	0.55	44.641	<0.001	0.025	
Q10							
	Main facility	2.124	1.05	4.064	0.044	8.364	[1.061–65.947]
	Constant	2.892	0.47	41.171	<0.001	19.723	
Q22							
	Main facility	−0.800	0.28	8.263	0.004	0.449	[0.260–0.775]
	Work experience >3 years	1.015	0.33	9.44	0.002	2.758	[1.444–5.269]
	Constant	−1.105	0.29	14.69	<0.001	0.331	
Q23							
	Main facility	−0.807	0.28	8.606	0.003	0.446	[0.260–0.765]
	Work experience >3 years	0.619	0.3	4.17	0.041	1.857	[1.025–3.363]
	Constant	0.640	0.25	6.356	0.012	1.896	
Q26							
	Main facility	−0.748	0.26	8.213	0.004	0.473	[0.284–0.790]
	Work experience >3 years	0.686	0.29	5.572	0.018	1.985	[1.123–3.507]
	Constant	0.049	0.25	0.04	0.841	1.05	

Q9—Have you ever had a videoconference with a healthcare professional as the patient? Q10—Do you have a device that allows you to conduct a videoconference (smartphone, computer, tablet)? Q22—I have easy access to in-person psychological and/or psychiatric care at the clinic. Q23—During a videoconference, I think I can spontaneously talk to my psychiatrist or psychologist about what worries me, even if they don’t ask. Q26—Videoconferences are safe and respect my privacy and medical confidentiality.

**Table A5:** Q14 bivariate and multivariate analysis: Chi-square and multinomial logistic regression.

Question No. 14	Category	Variable	Relative Frequency	Coefficient B	S.E	Wald	Sig	Exp(B)	95% CI of Exp(B)	Model Fit Sig (Final)	Pearson	Deviation	Nagelkerke R^2^ (Sig)	ROC Curve (AUC)
	Healthcare Unit	Main facility	23.2%	1.003	0.47	4.45	0.035	2.726	[1.073–6.923]	0.006	0.511	0.196	0.159	0.491
Option 2 *		Partner units	6.7%											
	Gender	Female	18.6%	1.191	0.46	6.67	0.010	3.292	[1.333–8.133]					0.594
		Male	9.6%											
Option 5 **	Gender	Female	44.9%	0.828	0.36	5.42	0.020	2.288	[1.140–4.593]					
		Male	36.9%											

Q14. What frequency do you consider appropriate for psychology/psychiatry videoconferences at your unit? * Annually, with additional sessions as needed. ** As requested by workers, at any time of the year.

**Table A6:** Validation results of the regression models.

Question No.	Omnibus Test (Model Chi-Square)	Omnibus Test (Model Sig)	Nagelkerke R^2^ (Sig)	Hosmer and Lemeshow Test (Sig)	Overall Accuracy	ROC Curve (AUC)
Q8 Clinicians vs. Administrative Staff	5.887	0.053	0.039	0.397	86.9%	0.587
Q9 Healthcare Unit	8.235	0.016	0.082	0.899	94.2%	0.318
Q10 Healthcare Unit	8.129	0.017	0.097	0.726	95.6%	0.331
Q15 Gender	10.83	0.004	0.068	0.112	85.5%	0.366
Q17 Age Group	12.43	0.002	0.141	0.964	88.6%	0.374
Q18 Gender	5.925	0.052	0.043	0.341	81.1%	0.596
Q19 Age Group	8.465	0.015	0.097	0.658	88.6%	0.411
Q22	15.483	<0.001	0.076	0.205	65.5%	
Healthcare Unit						0.57
Work Experience >3 years						0.429
Q23	10.64	0.005	0.053	0.909	67.6%	
Healthcare Unit						0.581
Work Experience >3 years						0.461
Q26	11.427	0.003	0.055	0.902	58.2%	
Healthcare Unit						0.572
Work Experience >3 years						0.453
Q27 Clinicians vs. Administrative Staff	10.683	0.005	0.054	0.351	70.9%	0.588

**Table A7:** Complementary analysis: Chi-square and binary logistic regression.

Question No.	Category	Relative Frequency (Yes)	X^2^ (Pearson’s Chi-Square)	df	*p*-Value of the Chi-Square	Regression Exp(B)	95% CI (Exp(B))
Q8							
	Professional Group: Administrative	94.4%	4.868	1	0.035	3.180	1.083–9.337
	Professional Group: Clinical	84.2%					
Q15							
	Female	93.2%	10.028	1	0.002	3.522	1.557–7.970
	Male	79.6%					
Q17							
	Age Group: 18–34	91.6%	19.128	1	<0.001	11.032	3.094–39.335
	Age Group: ≥50	50.0%					
Q18							
	Male	92.4%	4.015	1	0.045	2.135	1.003–4.714
	Female	84.7%					
Q19							
	Age Group: 18–34	91.0%	11.764	1	0.002	7.841	2.071–27.025
	Age Group: ≥50	58.3%					
Q27							
	Professional Group: Administrative	84.7%	9.022	1	0.004	2.859	1.412–5.790
	Professional Group: Clinical	66.0%					

Q8: If you had a psychological problem, would you feel comfortable doing a videoconference with a mental health professional? Q15: Have you ever felt an impact on your mental health due to your work? (Including stress, insomnia, anxiety, exhaustion, burnout)? Q17: During a videoconference, could I talk about my problems? Q18: During a videoconference, could I have a first consultation? Q19: During a videoconference, could I talk about myself? Q27: I feel sufficiently comfortable with the technology to do a videoconference.

## Appendix B

**Table A8:** Characterization of the interview participants.

Participant	Specialty	Average Age	Average Work Experience in the Network
1st Interviewee	Psychologist		
2nd Interviewee	Psychiatrist		
3rd Interviewee	Psychiatrist	42.1	23 years
4th Interviewee	Psychologist		
5th Interviewee	Psychologist		
