# Supplementary material for: Telemedicine as an Approach to the Mental Health of Healthcare Workers in Angola
Source: Int J Environ Res Public Health. 2025 Apr 4;22(4):565. doi: 10.3390/ijerph22040565 (PMC12027011; doi:10.3390/ijerph22040565)
Supplement: Supplementary file 1 [file ijerph-22-00565-s001.zip › Supplementary Materials File S1.pdf]

Supplementary Material 1.

1- Cultural Adaptation of the Questionnaire.

The questionnaire used for collecting quantitative data from professionals in the network in Angola was developed based on the instrument by Cormi C. et al. (2021), presented in the article "Telepsychiatry to Provide Mental Health Support to Healthcare Professionals during the COVID-19 Crisis." This choice was based on the unique opportunity offered by the study, considering the scarcity of research in this specific area. Furthermore, there was alignment in research objectives, as Cormi C. et al. (2021) sought to examine healthcare professionals' acceptance of a telepsychiatry service aimed at providing mental health support during the COVID-19 pandemic.

To use this questionnaire, the original authors were contacted via email, and permission was obtained for its use, as well as for its translation and cultural adaptation.

Cultural adaptation is an essential process when there is a linguistic and cultural discrepancy between the context of the questionnaire's origin and its intended application. This adaptation aims to ensure the semantic and conceptual equivalence of the questionnaire items. Consequently, the original questionnaire, written in English, was adapted into Portuguese following the guidelines established by Beaton et al. (2000) and Tsang et al. (2017). The cultural adaptation process was conducted in six distinct stages (see Figure S1).

The first phase of the process involved the direct translation of the questionnaire, carried out by two independent translators, both native Portuguese speakers. One translator was Angolan, and the other was Portuguese. Notably, one of the translators was not informed about the study's objectives, ensuring greater impartiality and linguistic adequacy during the translation process.

Next, the translated versions were synthesized, with specific adjustments made, particularly to the following questions: In question 7, "If you were facing psychological issues, would you feel comfortable having a video consultation with a mental health professional?", the term "comfortable" was replaced with "at ease" (à vontade), aiming for a better expression of the participant's feelings about having a video consultation with a mental health professional. In question 9, the English term "hardware/device" was changed to "material/dispositivo" in Portuguese for clearer comprehension. Additionally, the term "teleconsultation" was standardized to "videoconsultation" throughout the questionnaire to increase specificity and facilitate participants' understanding.

The adaptation of the original questionnaire to meet the objectives of the present study resulted in the addition of three questions, for demographics, and the modification of three others that did not align with the scope of the research.

The consensus-synthesized version was back-translated by an independent translator to revert the questionnaire to its original language. This procedure was carried out autonomously, without input from the Portuguese translators or access to the original text.

During the back-translation process, a combination of literal and interpretative approaches was adopted, with particular attention paid to correct grammar and syntax in English. No specific strategies were necessary for the translation, although specialized healthcare vocabulary was carefully considered. According to the back-translator, the translation process utilized word-processing software, working with a copy of the original text provided by the researcher. The back-translator performed a single revision after discussing contextual language usage with the researcher regarding question 3, particularly the term “other collaborator.” Notably, the back-translator had no access to the study objectives or knowledge of the research topic, ensuring the absence of conflicts of interest regarding the translation of the questionnaire items for this academic investigation.

The back-translated version was reviewed by one of the authors of the original article, identifying only a minor typographical error in the term “psychiatrist”.

Participation in the pilot test was voluntary. Two departments were randomly selected, where the researcher provided a tablet for participants to complete the questionnaire via Google Forms or to receive the link via WhatsApp. The completion time was recorded, and at the end of the questionnaire, verbal and written questions were posed regarding the comprehension of the items and the perception of the possible answers.

The pilot test proved essential for identifying difficulties in completing the online questionnaire. As a result, adjustments were made to facilitate the process, particularly in the formulation of questions with Likert Scales and in reorganizing demographic questions to the end of the questionnaire, aiming to capture respondents' attention with initial questions. Participants were informed that their participation in the pre-test would exclude them from participating in the final study.

The back-translated questionnaire received no evaluations from the authors that invalidated its semantic and conceptual equivalence compared to the original questionnaire.

In the final stage of cultural adaptation, a pilot test was conducted with a sample of participants possessing characteristics similar to the population providing care to patients. At this stage, a total of 32 responses were obtained, with 96.6% of participants considering the questionnaire easy to understand.

2- Questionnaire – Telepsychiatry for All

Thank you for your participation and interest in this study!

Demographic information

Q1. Age:

- 18–24 years old
- 25–34 years old
- 35–50 years old
- 50–60 years old
- 60 years old or above

Q2. Sex:

- Female
- Male

Q3. Occupation

- Doctor
- Nurse
- Technician (Pharmacy, Laboratory, Imaging/Radiology)
- Other Staff (Administrative and Receptionists)

Q4. Please select the region of the country where your unit is located:

- Capital Region (Luanda)
- Eastern Zone
- Southern Zone
- Northern Zone

Q5. How many years have you been working at the clinic?

- Less than 1 year
- 1 to 3 years
- More than 3 years

About teleconsultation

Q6. Have you ever sought help from a mental health professional for psychological or psychiatric reasons, for yourself and in person?

- Yes
- No

Q7. Have you ever used a video conferencing service (Skype, Teams, FaceTime, Messenger, House Party, WhatsApp, Hangout, Duo, etc.)?

- Yes
- No

Q8. If you were facing psychological issues, would you feel comfortable having a video consultation with a mental health professional?

- Yes
- No

Q9. Have you ever had a video consultation with a healthcare professional, with you being the patient?

- Yes
- No

Q10. Do you have tools/ devices that allows you to carry out a video consultation (smartphone, computer, tablet, etc.)?

- Yes
- No

Q11. Is your internet connection strong enough to hold a video conference (on your phone or at home)?

- Yes
- No

Q12. Do you have a space where you can be calm and speak freely for at least 30 minutes, should you have a video consultation?

- Yes
- No

Q13. Which of the following devices would you preferably choose for conducting video consultations?

- Smartphone
- Computer
- Tablet

Q14. What frequency do you consider appropriate for conducting psychology/psychiatry video consultations at your unit?

- Annually, during workers' health check-ups.
- Annually, with additional sessions as needed.
- Semiannually (every 6 months).
- Upon workers' request, at any time of the year.

Regarding psychiatric and/ or psychological support

Q15. Have you ever felt any impact on your mental health due to your work? (Including stress, sleep issues, anxiety, exhaustion, burnout...)

- Yes
- No

Q16. Faced with psychological issues, I think a video consultation would be:

- Easier than an in-person consultation
- Equivalent to a face-to-face consultation
- Harder than a face-to-face consultation
- I would not resort to a video consultation for psychological issues

Q17–21. During a teleconsultation, I could:

Talk about my problems:

- Yes, I could
- No, I could not

Do a first-time consultation:

- Yes, I could
- No, I could not

Talk about me:

- Yes, I could
- No, I could not

Being helped for a psychological issue:

- Yes, I could
- No, I could not

Accept a drug prescription:

- Yes, I could
- No, I could not

Relationship and communication

Please indicate your level of agreement with the following propositions:

Q22. I have easy access to in-person psychological and/ or psychiatric care:

- Strongly disagree
- Disagree
- Neither agree nor disagree
- Agree
- Strongly agree

Q23. During a video consultation, I think I can spontaneously talk to my psychiatrist or psychologist about what worries me, even if they don't ask me:

- Strongly disagree
- Disagree
- Neither agree nor disagree
- Agree
- Strongly agree

Q24. Through a video consultation, I think that my psychiatrist or psychologist can offer me the same quality of care as he or she would in person:

- Strongly disagree
- Disagree
- Neither agree nor disagree
- Agree
- Strongly agree

Q25. During a video consultation, I think that the doctor-patient relationship with a psychiatrist or psychologist would be satisfactory for me:

- Strongly disagree
- Disagree
- Neither agree nor disagree
- Agree
- Strongly agree

Q26. Video consultations are secure and respect my privacy and medical confidentiality:

- Strongly disagree
- Disagree
- Neither agree nor disagree
- Agree
- Strongly agree

Q27. I feel comfortable enough with technology to do a video consultation:

- Strongly disagree
- Disagree
- Neither agree nor disagree
- Agree
- Strongly agree

Q28. Considering my personal and professional limitations, a video consultation with a psychiatry or psychologist would allow me to organize myself more easily, compared to a face-to-face consultation:

- Strongly disagree
- Disagree
- Neither agree nor disagree
- Agree
- Strongly agree

Q29. I think that a video consultation with a psychiatrist is a useful tool to help healthcare professionals who have had to manage COVID-19 patients:

- Strongly disagree
- Disagree
- Neither agree nor disagree
- Agree
- Strongly agree

3- Details of Data Analysis and Processing.

Data analysis was performed using statistical methods with version 29 of the Statistical Package for the Social Sciences (SPSS). Since the questionnaire variables are predominantly nominal and ordinal categorical, absolute frequency and percentage were used for descriptive statistical analysis. Bivariate statistical analyses were conducted using the Chi-Square test, with Fisher's exact test applied when necessary. Statistical significance was considered at a 5% level ($P<0.05$).

Multivariate analysis was performed using binary logistic regression for questions with statistically significant values (8, 9, 10, 15, 17, 18, 19, 22, 23, 26, 27), with coding (0-No; 1-Yes) of the categorical dependent variables corresponding to the respective questionnaire questions with dichotomous responses. Odds ratios were adjusted for recoded variables such as gender, work experience, age group, and professional group, showing results similar to Chi-Square in questions 9 and 10.

In regression analysis, model adjustments were made by including all independent variables and removing them one by one based on the p-value. Models were considered valid when

statistically significant ($p<0.05$). Model fit quality was assessed using the Hosmer-Lemeshow test, which evaluates data adequacy to the model, with good fit indicated by non-significant results ($p>0.05$).

Predictive capacity of the models was evaluated using the area under the ROC (Receiver Operating Characteristic) curve, which expresses the discriminatory power of independent variables over dependents. Good predictive capacity was considered for AUC values above 0.7, while values between 0.5 and 0.7 indicated moderate to weak prediction.

Cox & Snell and Nagelkerke R^2 tests were used, although typically yielding modest values in regression models, to measure the proportion of variability in dependent variables explained by independents. Regression coefficients (Exp(B)) and respective 95% confidence intervals were calculated to interpret the effect of independent variables on dependents, identifying significant associations.

Multinomial logistic regression was conducted for question 14, due to the dependent variable having five categories. One category was chosen as the baseline, with others treated as dummy variables within the model. Likert scale questions were dichotomized to facilitate analysis into Agree (Strongly Agree and Agree) and Disagree/Neutral (Disagree, Strongly Disagree, Neutral).
