# Supplementary material for: Telemedicine as an Approach to the Mental Health of Healthcare Workers in Angola
Source: Int J Environ Res Public Health. 2025 Apr 4;22(4):565. doi: 10.3390/ijerph22040565 (PMC12027011; doi:10.3390/ijerph22040565)
Supplement: Supplementary file 1 [file ijerph-22-00565-s001.zip › Supplementary Materials File S2.pdf]

Supplementary Material 2

Interview Script.

Introduction and Context.

Thank you for participating in this interview. The objective of this research is to explore the perceptions of psychologists and psychiatrists regarding the future adoption of telemedicine within the healthcare network as a tool to support the mental health of healthcare professionals.

Your opinion will be highly valuable to this study. Please note that all information collected will be used solely for scientific purposes. There are no right or wrong answers.

Do you consent to participate in this study?

If yes, you must sign the informed consent form.

Questions:

Perception of Telemedicine Use

1. Have you ever conducted a video consultation to support the mental health of a patient?
2. How would you imagine the process of conducting a video consultation within the healthcare network?
3. In your perception, what would be the main advantages of video consultations for you as a mental health professional in Angola?
4. What would be the main advantages of video consultations for the workers within the network?
5. In your opinion, what challenges would be associated with using video consultations for the mental health of workers in the network?
6. Are there any specific situations where telemedicine could have a significant impact on your clinical practice?

Perception of Ease of Telemedicine Use

1. How do you evaluate the difficulty of using video consultations for the mental health of workers?
2. Which electronic devices do you prefer to use for video consultations regarding the mental health of workers?
3. What type of training or preparation would you consider necessary for the efficient use of video consultations?

4. What are your experiences and impressions of using video conferencing tools such as WhatsApp, Zoom, Skype, and Teams, especially in terms of ease or challenges encountered?

Intention to Use

1. What is your opinion about the possibility of implementing a video consultation service? Comment on its efficiency and its impact on patient relationships.
2. What factors would significantly influence your decision to adopt video consultations for mental health care?
3. How do you envision the role of implementing video consultations for mental health within the network in the coming years?
4. What actions would be necessary within the network to effectively integrate video consultations for workers' mental health?
5. In your opinion, what would be the ideal frequency for conducting video consultations for the mental health of workers?

Conclusion and Final Comments

Is there anything else you would like to add or highlight about telemedicine for the mental health of workers in the context of Angola?

Thank you for your participation in this interview.

Final Considerations.

Qualitative Results - Relevant segments of the interview

Questions:	Themes	Relevant segments of the interview
1. Have you ever conducted a video consultation to support the mental health of a patient?	Perceptions of Psychologists and Psychiatrists Regarding the Use of Telemedicine	"(...) “Three times a week, and I’ve been doing this for more than three years. Ah, since COVID, since the COVID pandemic started, I began doing video consultations. Since COVID, I have patients here, I have patients abroad, I have patients in the provinces, so I do this—it’s a routine medical practice.” (Interviewee 3, Pos. 24).<br><br>“I started individually following up with patients, including some who do not reside in Luanda or who, at a given time, traveled and always wanted to continue their follow-ups with me.” — Interviewee 2, Pos. 19
2. How would you imagine the process of conducting a video consultation within the healthcare network?		“(…) I think it would be very viable, in terms of privacy, for the worker to have their consultation in a confidential manner. If they are at work and available, or if they are not engaged in work activities and find a space to do it, they can proceed.” —Interviewee 1, Pos. 13<br><br>“I think the clinic adopting this would be an added value, but it could simply offer an alternative mechanism that provides continuity of care for our patients who, for some reason, live outside Luanda, or for any other reason find themselves outside Luanda or even outside the country and cannot physically travel to our facility. This way, we maintain their follow-up care.”- Interviewee 2, Pos. 39
3. In your perception, what would be the main advantages of video consultations for you as a mental health professional in Angola?		“(…) For some reason, patients missed their appointments or even have a schedule, but the schedule is empty. In this sense, it would also be possible to find a way to make productive use of that time.” — Interviewee 2, Pos. 55<br><br>“I once had a patient with psychomotor agitation who was not being managed in Benguela and had to be transported by ambulance. If we had conducted a consultation via WhatsApp, for example, especially with nursing assistance, the patient could have been managed easily without needing to come to Luanda.” — Interviewee 3, Pos. 41

		“One of the advantages I believe I would have if I conducted a video consultation with a patient is that I could possibly record the consultation and later review it. This might give me some insight into how the process went and perhaps help me improve in a future session.” — Interviewee 5, Pos. 14
4. What would be the main advantages of video consultations for the workers within the network?		“I always say that we need an organizational psychologist. People are referred to the clinical psychologist without being screened and without any input from the organizational psychologist. Because they start feeling unwell.. stress turns into anxiety, anxiety turns into depression, and depression turns into panic.” — Interviewee 1, Pos. 27<br><br>"In the context of mental health, it ends up being advantageous because the person, in the comfort of their home, in their privacy, can have a conversation with the doctor without the embarrassment of traveling, without the embarrassment of exposure, which is also a limitation for many. This ends up being very advantageous." — Interviewee 2, Pos. 58<br><br>"As often happens, the patient has to get on planes, pay for accommodations in Luanda, make appointment bookings, and then wait weeks due to a very large queue." — Interviewee 3, Pos. 60

5. In your opinion, what challenges would be associated with using video consultations for the mental health of workers in the network?		"Regarding the means as well, the quality of the internet, sometimes the person is speaking, the internet freezes, then suddenly it comes back, and during the time it comes back, the person is saying, 'Hello, doctor, hello, hello, hello,' and I'm also saying, 'So-and-so, so-and-so, are you there?'" — Interviewee 5, Pos. 40<br><br>"(…) We need availability for internet access, we need good, user-friendly technology that easily allows access and handling of the information." — Interviewee 2, Pos. 69<br><br>"(…) A platform or app that allows for some privacy, some confidentiality criteria with limits, or limiting access to clinical, administrative, and more social and demographic information of the patients." — Interviewee 2, Pos. 70<br><br>"(…) Also, sometimes we have to be honest. There are people who are not very familiar with technology. Sometimes, they may even feel anxious when told that it will be a video consultation, the anxiety will triple." — Interviewee 5, Pos. 42
6. Are there any specific situations where telemedicine could have a significant impact on your clinical practice?		"The online consultation is very demanding because of speech and non-verbal, facial or paraverbal language. Because you don't have the non-verbal language of your client, you become much more vigilant about any sign, any speech." — Interviewee 1, Pos. 60<br><br>"The mental health consultation is not like an orthopedic consultation, or like a surgical consultation. I don't necessarily need to touch the patient, I need to talk, to see, to see their facial expression, face, and voice. Nothing more. And at a distance, I do my work. If necessary, I request exams, the patient goes to the local laboratory, does the exams, sends them to me via WhatsApp, I review the exams, and if necessary, I refer the patient to another specialist. It's a huge advantage." — Interviewee 3, Pos. 44<br><br>"(…) And there are many times when, in a few minutes of contact, very pertinent situations can be clarified, because sometimes they would take days or even require much more logistics for someone to access an important detail, even a clinical detail that could impact the course of their condition. And in this sense, the impact is huge because things that seem

		minimal, but in contact with the attending physician, are clarified in minutes, which have a very big impact." — Interviewee 2, Pos. 82
1. How do you evaluate the difficulty of using video consultations for the mental health of workers?	**Perceptions of the Ease of Using Telemedicine**	"(…) I am willing to learn other apps, my mind is still open to learning." — Interviewee 1, Pos. 71<br><br>Interviewee 2: "I have used it regularly, without major issues, except for technical matters like internet, network, and signal quality, which sometimes can affect the consultation process, the quality of communication during the consultation, and aside from that, I don't see any significant difficulties." — Interviewee 2, Pos. 100
2. Which electronic devices do you prefer to use for video consultations regarding the mental health of workers?		"(…) I prefer a computer, without a doubt. We have a wider view for visualization, right? And I will have a better angle to read paraverbal cues." — Interviewee 1, Pos. 85.<br><br>"On a phone with good image and sound resolution, it's also possible. It's the simplest thing." — Interviewee 3, Pos. 116
3. What type of training or preparation would you consider necessary for the efficient use of video consultations?		"(…) A chapter of this training would have to address the importance of videoconsultations, it would have to select a series of elements that discuss the need for consultations in this format." — Interviewee 5, Pos. 59<br><br>"As I said, the app or platform has to be as user-friendly as possible. It has to be less, let's say, complicated, so that with 15 or 30 minutes of information, someone with the ability to handle an electronic device can enter, exit, input data, and then access the video and interact with the patient, right?" — Interviewee 2, Pos. 116<br><br>"I don't think any training is necessary. What is needed is to create a series of conditions, basic requirements that should be explained to the patients, what a videoconsultation is, and what needs to be done during a videoconsultation. So, it’s not about training, it’s about prerequisites that should be written down. The patient, before having the consultation, will be informed, will sign, and then the consultation takes place." — Interviewee 3, Pos. 122
4. What are your experiences and impressions of using video conferencing tools such as WhatsApp, Zoom, Skype,		"Then, we set a time with the patient, and I do these consultations individually, let's say, I end up defining the end of the day, when I'm already at home, to provide the follow-up, and the consultations have

and Teams, especially in terms of ease or challenges encountered?		been like this. WhatsApp is the most user-friendly and the most available in the patients' scenario." — Interviewee 2, Pos. 126
1. What is your opinion about the possibility of implementing a video consultation service? Comment on its efficiency and its impact on patient relationships.	**Professionals' Intentions to Use Telemedicine**	"I don't think there needs to be an independent service. I believe that, for efficiency, what should be considered is integrating this into the overall activities of the mental health department." — Interviewee 2, Pos. 135<br><br>"Look, currently, more young people go to psychology consultations, and young people prefer virtual environments rather than probably a more formal office, which can be more overwhelming for them, so there's that advantage too." — Interviewee 4, Pos. 99<br><br>"(…) On the other hand, for workers, it should also improve the satisfaction of our clients." — Interviewee 3, Pos. 135<br><br>"There must be all the requirements, all the prerequisites, so that it is a medical act carried out within the ethical and deontological principles of medicine." — Interviewee 3, Pos. 138
2. What factors would significantly influence your decision to adopt video consultations for mental health care?		"A good administration of this service, fees, that's important. I will be paid for my time available for these online consultations. If the patient misses the appointment, it's no longer my problem." — Interviewee 1, Pos. 125<br><br>"I could attend to a larger number of people. I could have a longer schedule and be able to see patients in the morning, afternoon, or even at night, just like a bank, right?" — Interviewee 4, Pos. 113<br><br>"The rest is the same as consultations; if they call me to say that there are patients who need to be seen, I will go. It's much more about the fact that there are patients who need our services and the care we can offer, and that's enough for me. If there are patients in need and I can help, I do it." — Interviewee 2, Pos. 148
3. How do you envision the role of implementing video consultations for mental health within the network in the coming years?		"I see this, hmmm, in a global context, I think it's inevitable, it's something that, whether we want it or not, we'll have to think about. Technology is evolving, it seems to me that there are many things we need to do, we can't shut ourselves off and ignore the trend of how things are on an international level." — Interviewee 2, Pos. 150

		"(…) For the follow-up of professionals, I think it's very well understood and welcome, and this is because we have a network, which is expanding widely across the country, and we need a strategy that ends up requiring this mental health support." — Interviewee 2, Pos. 152<br><br>"Not only at our network, we also have other networks, and if we can be close to our... even from a distance, I think it will be an added value." — Interviewee 2, Pos. 152
4. What actions would be necessary within the network to effectively integrate video consultations for workers' mental health?		"First, we need someone who cares about it. The people who are bothered have to know how to show what the clinic has been losing because of it. Once the clinic sees what is being lost, there will be attention." — Interviewee 5, Pos. 110<br><br>"(…) It is necessary to make a written proposal to the president of the board of directors." — Interviewee 3, Pos. 155<br><br>"Anyway, the creation of a platform that could unite users and healthcare professionals with a very accessible system, that isn't very bureaucratic, that doesn't have too many windows to fill in many fields, but a simple and accessible system." — Interviewee 5, Pos. 114<br><br>"(…) Train the people who will manage this follow-up model, both in terms of administrative management of data and in terms of managing sensitive patient data." — Interviewee 2, Pos. 156<br><br>"(…) And then the network needs to invest, because everything that comes next is investment. Electronic equipment is investment, maintenance of all equipment, system, and internet are investments. So, the biggest highlight for me would be the investment." — Interviewee 5, Pos. 112<br><br>"I think it needs to be well understood. Well, this app, this well-organized management, with all its features and variables, right? That we can really modulate, right? For the different situations that may arise, we need to have the resources to handle them and not say, 'Oh, it's taking too long here, taking too long there.' No, no. It has to be organized, with good organization." — Interviewee 1, Pos. 155

5. In your opinion, what would be the ideal frequency for conducting video consultations for the mental health of workers?		“Now, if we talk about frequency, for example, how the weekly schedule would be organized, the issue of workload distribution per professional throughout the week, this should align with in-person consultations and managing a significant number of patients. At least until we can promote it in a way that people also feel comfortable engaging in this virtual follow-up.” — Interviewee 2, Pos. 166<br><br>“(…) depending on the demand as well, right? If there is more demand, I will allocate more time.” — Interviewee 1, Pos. 163
	**Perceptions of Mental Health in Angola**	"People don't have the culture of seeing a psychologist to ask for help, which is essential, help, help. It seems like we're forbidden to have depression, anxiety, panic syndrome, schizophrenia, delusional disorder, or even alcohol or drug dependency. We're forbidden." — Interviewee 1, Pos. 172<br><br>"Employees will only function if they have mental health, because mental disorders compromise functionality at work." — Interviewee 5, Pos. 126<br>"(…) There can be high losses, from misuse of materials, work accidents involving the patient, even leading to the patient's death." — Interviewee 5, Pos. 126<br><br>"A depressed employee goes to work, but they're no longer working. They think they’re working, but they’re just going to work." — Interviewee 5, Pos. 131<br><br>"When working in a hospital unit, it's very visible that people are afraid to go for a consultation, mainly due to criticism from colleagues." — Interviewee 1, Pos. 171<br><br>"A mentally healthy worker can easily get motivated, and when well-motivated, they produce a lot. (…) The company is always ahead of problems, not behind. So, the money the company doesn't invest more in mental health prevention, it’s actually losing." — Interviewee 5, Pos. 135
